# Histone methyltransferase MLL1 drives renal tubular cell apoptosis by p53-dependent repression of E-cadherin during cisplatin-induced acute kidney injury

**DOI:** 10.1038/s41419-022-05104-0

**Published:** 2022-09-06

**Authors:** Chunyun Zhang, Yingjie Guan, Jianan Zou, Xu Yang, Georgia Bayliss, Shougang Zhuang

**Affiliations:** 1 Department of Medicine, Rhode Island Hospital and Alpert Medical School, Brown University, Providence, RI R02903 USA; 2 Department of Nephrology, Wuhan Union Hospital, Huazhong University of Science and Technology, Wuhan, China; 3 Department of Nephrology, Shanghai East Hospital, Tongji University School of Medicine, Shanghai, China

**Keywords:** Acute kidney injury, Methylation

## Abstract

Mixed lineage leukemia 1 (MLL1) is a histone H3 lysine 4 (H3K4) methyltransferase that interacts with WD repeat domain 5 (WDR5) to regulate cell survival, proliferation, and senescence. The role of MLL1 in the pathogenesis of acute kidney injury (AKI) is unknown. In this study, we demonstrate that MLL1, WDR5, and trimethylated H3K4 (H3K4me3) were upregulated in renal tubular cells of cisplatin-induced AKI in mice, along with increased phosphorylation of p53 and decreased expression of E-cadherin. Administration of MM102, a selective MLL1/WDR5 complex inhibitor, improved renal function and attenuated tubular injury and apoptosis, while repressing MLL1, WDR5, and H3K4me3, dephosphorylating p53 and preserving E-cadherin. In cultured mouse renal proximal tubular cells (RPTCs) exposed to cisplatin, treatment with MM102 or transfection with siRNAs for either MLL1 or WDR5 also inhibited apoptosis and p53 phosphorylation while preserving E-cadherin expression; p53 inhibition with Pifithrin-α lowered cisplatin-induced apoptosis without affecting expression of MLL1, WDR5, and H3K4me3. Interestingly, silencing of E-cadherin offset MM102’s cytoprotective effects, but had no effect on p53 phosphorylation. These findings suggest that MLL1/WDR5 activates p53, which, in turn, represses E-cadherin, leading to apoptosis during cisplatin-induced AKI. Further studies showed that MM102 effectively inhibited cisplatin-triggered DNA damage response (DDR), as indicated by dephosphorylation of ataxia telangiectasia mutated (ATM) and ATM and Rad-3 related (ATR) proteins, dephosphorylation of checkpoint kinase 1 and 2 (Chk1 and Chk2); depression of γ-H2AX; and restrained cell cycle arrest, as evidenced by decreased expression of p21 and phospho-histone H3 at serine 10 in vitro and in vivo. Overall, we identify MLL1 as a novel DDR regulator that drives cisplatin-induced RPTC apoptosis and AKI by modulating the MLL1/WDR5-/ATR/ATM-Chk-p53-E-cadherin axis. Targeting the MLL1/WDR5 complex may have a therapeutic potential for the treatment of AKI.

## Introduction

DNA damage and DNA damage response (DDR) play a critical role in cisplatin-associated acute kidney injury (AKI). Upon injection, cisplatin accumulates in renal tubular cells to trigger inter- and intra-strand DNA crosslinks and perturb DNA replication and transcription, which eventually causes cell cycle arrest and apoptosis. Multiple DDR pathways have been reported to be altered in cisplatin-induced AKI with ataxia telangiectasia mutated (ATM) and ATM and Rad-3-related (ATR) kinases being two key upstream regulators of cellular response to DNA damages [1].

Phosphorylation of the histone variant H2AX at serine 139, forming γ-H2AX, is an early cellular response to the induction of DNA double-strand breaks and associates with phosphorylation of ATR and ATM [2]. Activated H2AX can help ATR and ATM to phosphorylate Chk1 and Chk2 (the cell-cycle checkpoint kinase), leading to p53 phosphorylation. It has been documented that ATR/Chk2/p53 signaling mediates early DDR;[3] ATM/Chk1/p53 may mediate DNA repair in the early phase of cisplatin treatment, but it facilitates apoptosis at the late stage.

The p53-dependent pathway is critically implicated in DDR [4–6]. p53 can transcriptionally induce expression of apoptotic genes Bax and PUMA-α (p53 upregulated modulator of apoptosis-α), leading to caspase-3 activation and cell death. It can increase expression of p21, resulting in senescence/cell cycle arrest [1, 7–9]. On the other hand, p53 is also necessary for regulating the expression of E-cadherin, a key adhesion molecule maintaining renal epithelial cell (REC) integrity [10, 11]. Loss of E-cadherin often results in cell detachment from extracellular matrix [12, 13]. Our recent study has shown that damage of REC integrity by downregulation of E-cadherin promotes apoptosis [14].

DDR and E-cadherin loss are subjected to regulation by multiple mechanisms, including epigenetic modifications [12, 15]. Mixed lineage leukemia 1 (MLL1) has been recognized as a key histone H3 lysine 4 methyltransferase [16]. Activation of MLL1 requires it to form a complex with WD repeat-containing protein 5 (WDR5) [17–19]. Pharmacological interruption of the MLL1-WDR5 interaction leads to MLL1 inactivation, and this approach has been used as a therapeutic strategy for treatment of various tumors [17–19]. Inhibition of the MLL1/WDR5 complex can also protect against renal senescence, inflammation, and fibrosis in an ischemia/reperfusion mouse model [20]. The role of the MLL1/WDR5 complex in AKI remains unknown.

We investigated the role of MLL1 in cisplatin-induced AKI in vivo and in vitro. Our results demonstrate that MLL1, in complex with WDR5, is activated following cisplatin treatment, and engages in the early response to DNA damage, leading to renal tubular cell apoptosis by p53-dependent repression of E-cadherin.

## Results

### MM102 attenuates AKI after cisplatin administration in mice

**Fig. 1:**
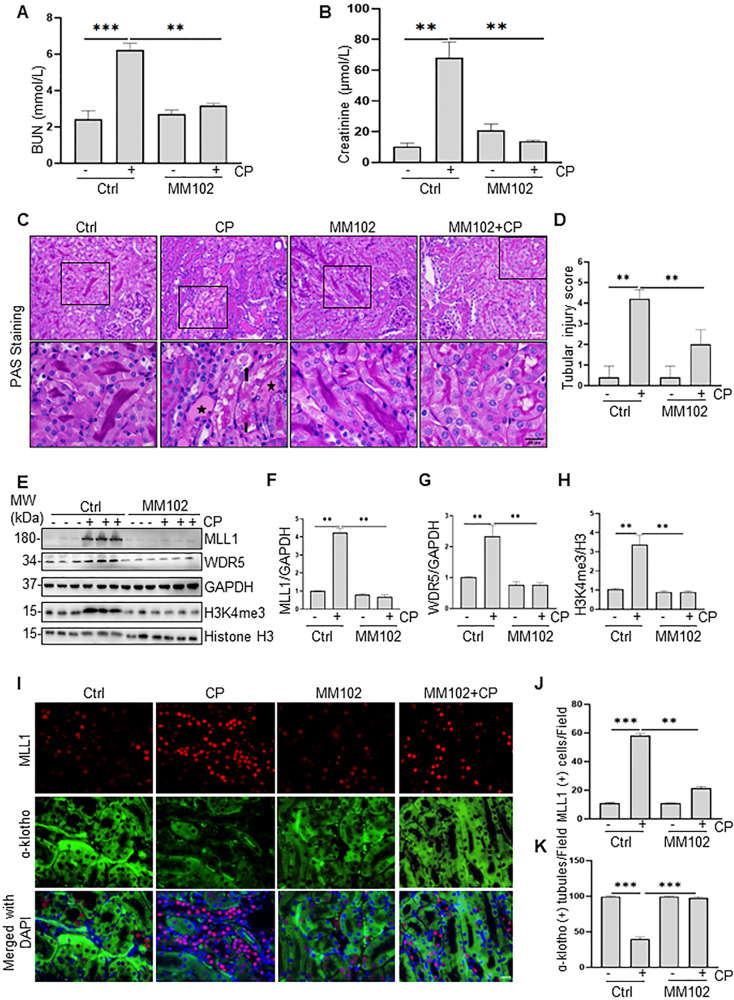
Pharmacological inhibition of MLL1/WDR5 activity with MM102 improves renal function and attenuates renal pathology in cisplatin (CP)-induced acute kidney injury (AKI). Mice in the group of CP and MM102 plus CP were intraperitoneally injected with CP at the dose of 20 mg/kg. MM102 (15 mg/kg) was administered intraperitoneally 2 h before the CP injection and then given daily for three consecutive days. All the mice were euthanized 72 h after CP injection. Blood samples and kidney tissues were collected for further analysis. **A**, **B** Blood urea nitrogen (BUN) and serum creatinine (Scr) were detected as measure of kidney function (*n* = 6 for each group). **C**, **D** Representative sections of Periodic acid-Schiff (PAS) staining of kidney tissues (magnification ×200, ×600, respectively). Arrows indicate cell shedding and tubular atrophy; and asterisks indicate cast formation. Scale bars: white 50 μm, black 20 μm. The degree of tubular injury was scored by the method as described in the “Materials and Methods” (*n* = 6 for each group). **E**–**H** Kidney lysates were subjected to immunoblot analysis using antibodies against MLL1, WDR5, and H3K4me3; GAPDH and histone H3 were used as loading control (*n* = 6 for each group). Expression levels of MLL1 (**F**), WDR5 (**G**), and H3K4me3 (**H**) were quantified by densitometry and normalized with GAPDH and histone H3, respectively. **I** Immunofluorescent staining for MLL1 and ɑ-klotho is shown (*n* = 6 for each group). Scale bars = 20 μm. **J**, **K** Quantification of cells with positive staining of individual proteins per field as indicated. All control samples shown were from three independent mice. After normalization with references, signal of control samples was arbitrarily set as 1, and the signals of other lanes were normalized with control to calculate fold changes. Values are mean ± SD. **P < 0.01, ***P < 0.001.

To investigate the role of MLL1/WDR5 in cisplatin-induced AKI, mice were treated with MM102, an inhibitor of the MLL1/WDR5 complex [21], or vehicle 2 h before cisplatin administration (20 mg/kg, intraperitoneally injection). MM102 was then given daily for three consecutive days. Blood samples and kidney tissue were collected 72 h after cisplatin injection. Blood urea nitrogen (BUN) and serum creatinine (SCr) were used as measures of renal function. As shown in Fig. 1A, BUN levels in cisplatin group were much higher than that in control group (6.217 ± 0.374 vs. 2.420 ± 0.470 mmol/L) (***P < 0.001); MM102 treatment reduced the cisplatin-boosted BUN to 3.172 ± 0.114 mmol/L (**P < 0.01). Similarly, SCr was 68.126 ± 10.217 μmol/L in cisplatin-alone group (Fig. 1B), higher than that in the control group (10.322 ± 2.135 μmol/L) (**P < 0.01); MM102 treatment significantly reduced SCr to 20.922 ± 4.016 μmol/L (**P < 0.01); MM102 alone had little effect on either BUN or SCr.

Periodic acid-Schiff (PAS) staining showed cisplatin-induced kidney damage, as indicated by dilated tubules with cast formation, cell shedding, and tubular atrophy. MM102 administration significantly ameliorated these pathological changes (Fig. 1C). There were no significant pathological changes in the vehicle-operated kidney with or without MM102 treatment. The pathological score was calculated by averaging the grades assigned to tubules in all fields (Fig. 1D; **P < 0.01).

Cisplatin administration boosted the expression of MLL1, WDR5, and trimethylated histone H3 lysine 4 (H3K4me3). MM102 treatment efficiently inhibited the expression of MLL1 and WDR5 as well as its methyltransferase activity, marked by decreased H3K4me3 levels (Fig. 1E–H). Immunofluorescence (IF) staining also verified that MM102 reduced cisplatin-associated elevation of MLL1 and restored cisplatin-induced downregulation of ɑ-klotho (a renoprotective marker) [22]. Notably, upregulation of MLL1 and downregulation of ɑ-klotho overlapped in renal proximal tubular cells (RPTCs) (Fig. 1I–K).

These results illustrate that MLL1/WDR5 plays a critical role in cisplatin-induced AKI and that MM102 is potentially renal protective.

### MM102 reduces apoptosis, along with reduced p53 phosphorylation and retained E-cadherin expression in vivo

**Fig. 2:**
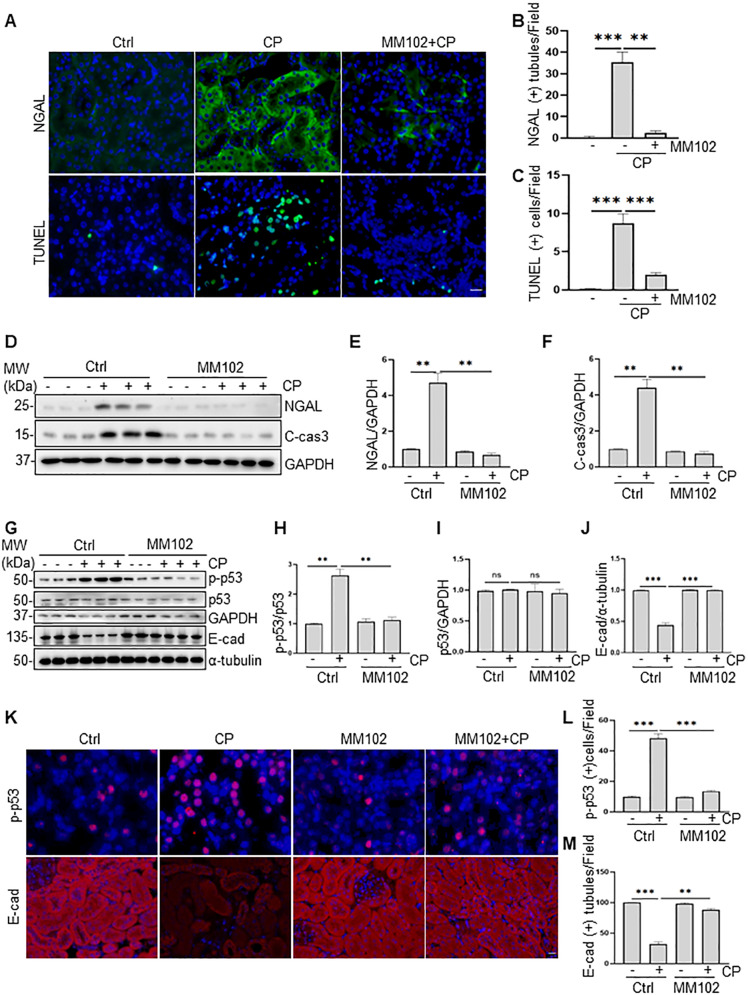
MM102 reduces renal tubular injury and apoptosis, decreases expression of p-p53, and restores E-cadherin (E-cad) in the kidney of mice following cisplatin (CP) treatment. Mice in the group of CP and MM102 plus CP were intraperitoneally injected with CP at the dose of 20 mg/kg. MM102 (15 mg/kg) was administered intraperitoneally 2 h before the CP injection and then given daily for 3 consecutive days. All the mice were euthanized 72 h after CP injection. **A** Kidney tissue was collected and subjected to neutrophil gelatinase-associated lipocalin (NGAL) fluorescent staining and TdT-mediated dUTP nick-end labeling (TUNEL) staining. **B**, **C** Fluorescent intensity of NGAL per field and the number of TUNEL-positive cells per high power field (*n* = 6 for each group) were accounted and averaged. Scale bar = 20 μm. **D** Kidney tissue lysates were subjected to immunoblot analysis with antibodies against NGAL or caspase3-cleavage (C-cas3); GAPDH was used as loading control. Expression levels of NGAL (**E**) and C-cas3 (**F**) were quantified by densitometry and normalized with GAPDH. Kidney tissue lysates were subjected to immunoblot analysis with antibodies against phospho-p53 (p-p53), p53, or E-cad (**G**). GAPDH and α-tubulin were used as loading controls. Expression levels of p-p53 (**H**), p53 (**I**), and E-cad (**J**) were quantified by densitometry and normalized with GAPDH or α-tubulin or total p53 as indicated. **K** Immunofluorescent staining for p-p53 and E-cad is shown. Scale bars = 20 μm. Quantification of cells with positive staining for p-p53 per field (**L**) or E-cad positive area per field (**M**) is shown. All control samples shown were from three independent mice. After normalization with reference, signal of control samples was arbitrarily set as 1, and the signals of other lanes were normalized with control to calculate fold changes. Values are mean ± SD. A representative result from at least three experiments was shown. *P < 0.05, **P < 0.01, ***P < 0.001.

IF staining indicated that neutrophil gelatinase-associated lipocalin (NGAL, an early biomarker of AKI) was increased in kidneys exposed to cisplatin relative to sham-operated kidneys. Administration of MM102 dramatically reduced NGAL expression in cisplatin-injured kidneys (Fig. 2A, B). Consistently, TdT-mediated dUTP-X nick-end labeling (TUNEL) staining displayed increased number of apoptotic cells in injured kidney and MM102 largely inhibited this response (Fig. 2A, C). Moreover, increased expression of NGAL and cleavage of caspase-3 (C-cas3, a recognized marker of apoptosis) in the kidney after cisplatin administration were detected by immunoblot analysis; treatment with MM102 returned these changes to base levels (Fig. 2D–F).

We further demonstrated that cisplatin increased p53 phosphorylation at serine 15 (~1- to 2-fold increase) and simultaneously reduced E-cadherin expression (~1-fold decrease); MM102 treatment repressed phosphorylated p53 (p-p53) to the basal level, but fully preserved E-cadherin expression (Fig. 2G–J). IF staining showed that p-p53 was mainly located in the nucleus and E-cadherin was in the cell membrane (Fig. 2K–M). Cisplatin treatment increased p53 phosphorylation and decreased E-cadherin expression. Administration of MM102 reversed these changes. These data suggest that protection against AKI conferred by MM102 may be associated with inactivation of p53 and preservation of E-cadherin in the cisplatin-damaged kidney.

### MM102 attenuates cisplatin-induced apoptosis in RPTCs

**Fig. 3:**
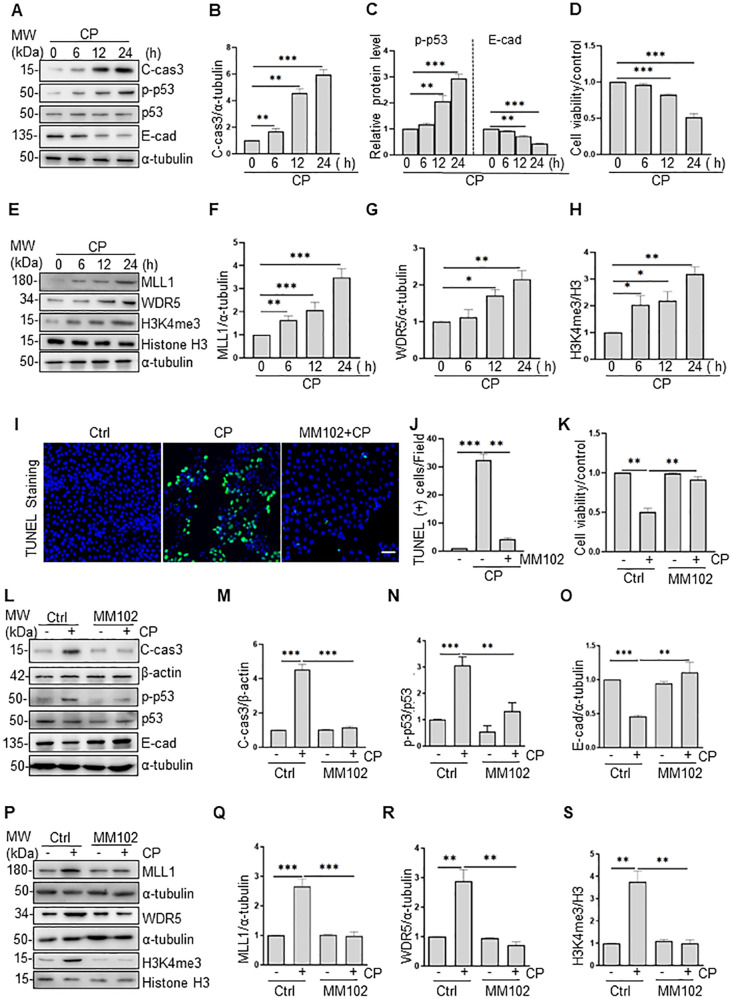
MM102 attenuates apoptosis, reduces p53 phosphorylation, and retains E-cadherin (E-cad) expression in cultured murine renal proximal tubular cells (RPTCs) exposed to cisplatin (CP). RPTCs were treated with CP (20 μM) with or without MM102 (50 μM) for 0 h, 6 h, 12 h, and 24 h, respectively (**A**, **E**) and 24 h (**L**, **P**). Cell lysates were prepared and subjected to immunoblot analysis with antibodies against cleaved caspase3 (C-cas3), phospho-p53 (p-p53), p53, and E-cad (**A**, **L**) and MLL1, WDR5, H3K4me3 (**E**, **P**) as indicated. Expression levels of all those proteins were quantified by densitometry; C-cas3 (**B**, **M**), and E-cad (**C**, **O**), MLL1 (**F**, **Q**), WDR5 (**G**, **R**) were normalized with α-tubulin or β-actin; p-p53 (**C**, **N**) was normalized with total p53. H3K4me3 (**H**, **S**) was normalized with histone H3. Signal obtained from the control (Ctrl) sample was set as 1. RPTCs were treated as indicated in “Materials and methods”. **D**, **K** Cell viability was detected 24 h after CP administered by cell counting kit 8 (CCK8) assay. **I**, **J** TUNEL staining was conducted 24 h after CP administered. TUNEL-positive cells were calculated and shown by at least 10 fields per section. Scale bar = 20 μm. Values are mean ± SD. Each representative blot from at least three experiments was shown. *P < 0.05, **P < 0.01, ***P < 0.001.

Exposure of RPTCs to cisplatin resulted in enhanced cell apoptosis, as measured by C-cas3 (Fig. 3A, B), and impaired cell viability measured by cell counting kit 8 (CCK8) assay (Fig. 3D), both of which occurred in a time-dependent manner. Cisplatin treatment also induced a time-dependent increase of p-p53 (Fig. 3A, C left) as well as decease of E-cadherin (Fig. 3A, C right), and increase of MLL1, WDR5, and H3K4me3 (Fig. 3E–H). Pretreatment with MM102 ameliorated cisplatin-induced apoptosis, as represented by reduced TUNEL-positive cells (Fig. 3I, J), and improved cell viability (Fig. 3K, Supplemental Fig. 1C) and C-cas3 (Fig. 3L, M, Supplemental Fig. 1A, B). In addition, MM102 administration restored the loss of E-cadherin to a base level after cisplatin treatment (Fig. 3L, O).

Meanwhile, p-p53 (ser15) increased 6 h after cisplatin exposure and continued to rise 3-fold in 24 h compared to control (Fig. 3A, C left); MM102 treatment reduced p-p53 (ser15) level by half compared to cisplatin alone (Fig. 3L, N). IF staining showed that p-p53 (ser15) overlapped with 4’,6’-diamidino-2-phenylindole dihydrochloride (DAPI), a nuclear stain (Supplemental Fig. 2A); MM102 decreased the number of cisplatin-induced p-p53 (ser15) positive cells (Supplemental Fig. 2B). MM102 lowered the level of MLL1, WDR5, and H3K4me3 in RPTCs exposed to cisplatin (Fig. 3P–S).

To further investigate the interactions between MLL1 and WDR5, we conducted co-immunoprecipitation for MLL1 and WDR5 in RPTCs with or without cisplatin treatment. As shown in Supplemental Fig. 3A, B, MLL1 and WDR5 could pull down each other by an antibody against each of them, indicating there exists a direct interaction between MLL1 and WDR5.

Collectively, blocking MLL1/WDR5 notably inhibited cisplatin-induced apoptosis in association with reduced p53 phosphorylation and preservation of E-cadherin.

### MLL1 or WDR5 knockdown attenuates cisplatin-induced apoptosis, along with reduced p53 phosphorylation and preserved E-cadherin in vitro

**Fig. 4:**
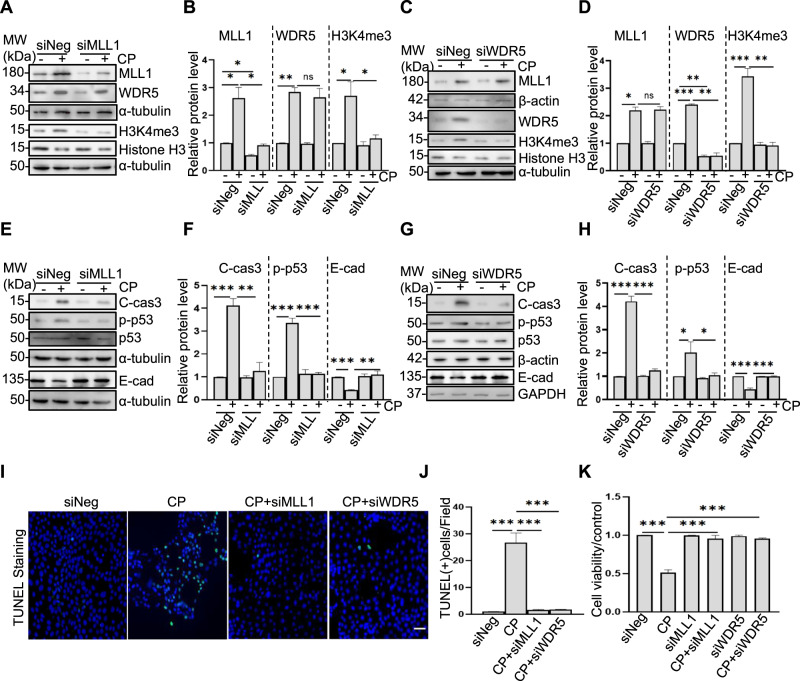
Silencing of MLL1 and WDR5 inhibits apoptosis, reduces p53 phosphorylation, and retains E-cadherin (E-cad) expression in cultured murine renal proximal tubular cells (RPTCs) exposed to cisplatin (CP). Cultured RPTCs were transfected with negative control siRNA (siNeg) or specific siRNA to MLL1 (siMLL1) or WDR5 (siWDR5) and then exposed to CP for another 24 h as described in “Materials and Methods”. **A**, **C**, **E**, **G** Cell lysates were prepared and subjected to immunoblot analysis with antibodies against MLL1, WDR5, H3K4me3, cleaved caspase3 (C-cas3), phospho-p53 (p-p53), p53, and E-cad as indicated. Expression levels of all those proteins were quantified by densitometry. MLL1 (**B**, **D** left) and WDR5 (**B**, **D** middle) were normalized with α-tubulin and β-actin, respectively; H3K4me3 (**B**, **D** right) was normalized with histone H3. C-cas3 (**F**, **H** left) was normalized with α-tubulin or β-actin, p-p53 (**F**, **H** middle) was normalized with total p53, and E-cad (**F**, **H** right) were normalized with α-tubulin or β-actin, respectively. **I**, **J** TUNEL staining was conducted 24 h after CP administered, and TUNEL-positive cells were calculated and shown by at least 10 fields per section. Scale bar = 20 μm. **K** Cell viability was detected by cell counting kit 8 (CCK8) assay. Signal obtained from the control (Ctrl) sample was set as 1. Each representative blot from at least three experiments was shown. Values are mean ± SD. *P < 0.05, **P < 0.01, ***P < 0.001. ns means not significant.

To validate the role of the MLL1/WDR5 complex in cisplatin-induced apoptosis of RPTCs, we further examined the effect of MLL1 or WDR5 knockdown on caspase-3 cleavage, apoptosis, and cell viability. Transfection of RPTCs with specific small interfering RNA (siRNA) either to MLL1 or WDR5 resulted in a significant downregulation of MLL1 (Fig. 4A, B left) or WDR5 (Fig. 4C, D middle), respectively, along with diminished expression of H3K4me3 (Fig. 4A, B right) (Fig. 4C, D right). Transfection with either MLL1 siRNA or WDR5 siRNA also reduced expression of cleaved caspase-3 (Fig. 4E, F left) (Fig. 4G, F left), and TUNEL-positive cells (Fig. 4I, J), and enhanced cell viability (Fig. 4K). Consistent with previous results obtained using MM102, MLL1, or WDR5 silencing decreased p-p53 (Fig. 4E, F middle) (Fig. 4G, H middle) and restored E-cadherin expression (Fig. 4E, F right) (Fig. 4G, H right) in cisplatin-treated RPTCs. Notably, siMLL1 had no effect on the expression of WDR5; and siWDR5 rarely affected the expression of MLL1 (Fig. 4A–D).

These results confirm the involvement of MLL1/WDR5 in the pathogenesis of cisplatin nephrotoxicity via a mechanism associated with p53 phosphorylation and E-cadherin downregulation.

### E-cadherin silencing counteracts the protective effect of MM102 in RPTCs following cisplatin exposure

**Fig. 5:**
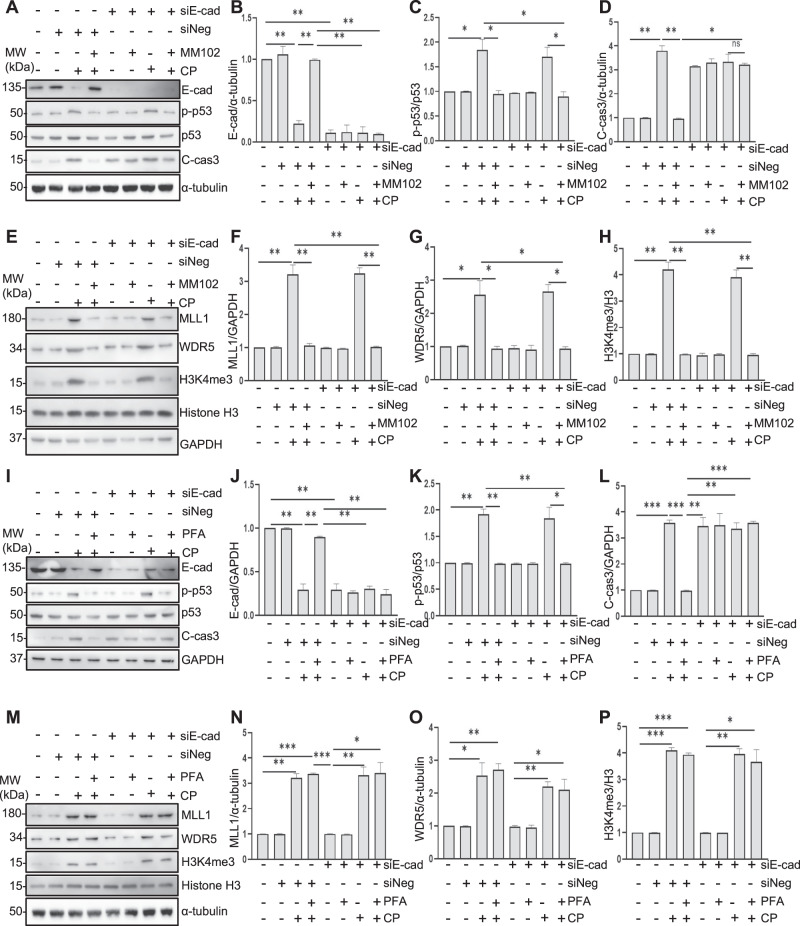
E-cadherin (E-cad) silencing reduces the protective effect of MLL1/WDR5 inhibition in murine proximal tubular epithelial cells (RPTCs) exposed to cisplatin (CP). RPTCs were transfected with siRNA targeting E-cadherin (siE-cad) or negative siRNA (siNeg) and then treated with MM102 (50 μM) or PFA (10 μM) for 1 h and then exposed to CP (20 μM) for an additional 24 h. **A**, **I** Cell lysates were prepared and subjected to immunoblot analysis with antibodies against E-cad, phospho-p53 (p-p53), p53, cleaved caspase3 (C-cas3), and α-tubulin. Expression levels of all those proteins were quantified by densitometry and E-cad (**B**, **J**), C-cas3 (**D**, **L**) were normalized with α-tubulin or GAPDH. p-p53 (**C**, **K**) was normalized with p53. **E**, **M** Cell lysates were prepared and subjected to immunoblot analysis with antibodies against MLL1, WDR5 and H3K4me3, and GAPDH/α-tubulin. Expression levels of all those proteins were quantified by densitometry and MLL1 (**F**, **N**), WDR5 (**G**, **O**) were normalized with GAPDH/α-tubulin. H3K4me3 (**H**, **P**) was normalized with histone H3. Values are mean ± SD. Signal obtained from the control (Ctrl) sample was set as 1. A representative result from at least three experiments was shown. *P < 0.05, **P < 0.01, ***P < 0.001.

We examined whether E-cadherin preservation would be necessary for the anti-apoptotic effect of MM102 in cisplatin-induced RPTCs apoptosis. Transfection with E-cadherin siRNA (siEcad) significantly reduced E-cadherin protein levels (Fig. 5A, B), restored expression of cleaved caspase 3, and diminished the anti-apoptotic effect of MM102 in RPTCs exposed to cisplatin (Fig. 5A, D). However, siEcad did not affect expression of p-p53 in the presence of MM102 under cisplatin exposure (Fig. 5A, C). In addition, Fig. 5E–H showed that siEcad had no effect on the expression of MLL1, WDR5, and H3K4me3, suggesting that E-cadherin may act downstream of MLL1/WDR5 to rescue cisplatin-exposed RPTCs from apoptosis.

To further explore the relation between E-cadherin and p53 in cisplatin-induced tubular cell apoptosis, we investigated the effect of Pifithrin-α (PFA, a p53 inhibitor) on cisplatin-induced caspase 3 cleavage in E-cadherin-silenced RPTCs. Transfection of E-cadherin siRNA significantly reduced E-cadherin protein levels (Fig. 5I, J), contributed to caspase 3 cleavage and diminished the anti-apoptotic effect of PFA in RPTCs following cisplatin treatment (Fig. 5I, L). siEcad did not affect expression of p-p53 and total p53 with/without PFA in the presence of cisplatin (Fig. 5I, K). Silencing E-cadherin and/or PFA had no effect on expression of MLL1/WDR5 and H3K4me3 (Fig. 5M–P).

These data illustrated that preservation of E-cadherin is critical for MM102 to confer its renoprotective effect, while E-cadherin is not essential for the cisplatin-induced regulation of MLL1/WDR5 and p53. This suggests that E-cadherin acts downstream of MLL1/WDR5 and p53.

### Knockdown and inhibition of p53 restore E-cadherin expression and decrease apoptosis in vitro

**Fig. 6:**
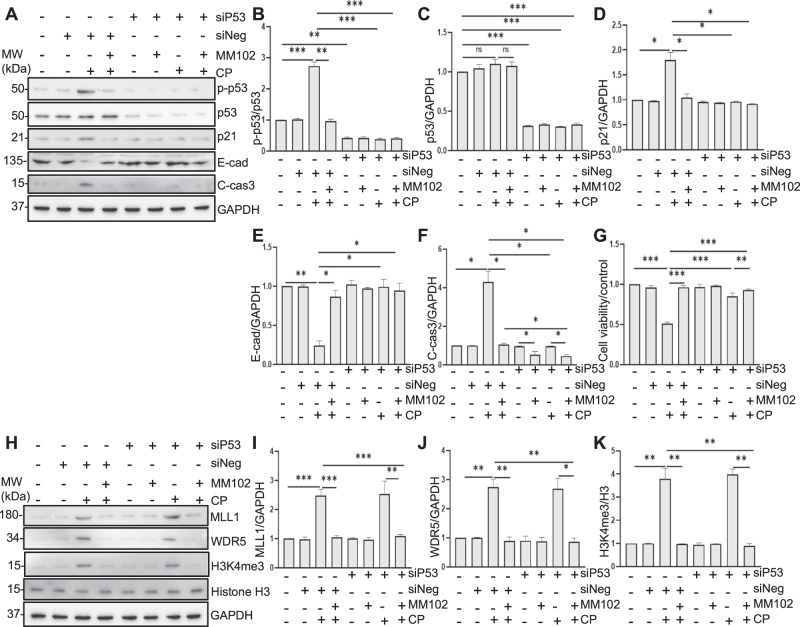
Knockdown of p53 with silence RNA (siRNA) diminishes cisplatin (CP)-induced apoptosis along with preservation of E-cadherin (E-cad) levels in murine renal proximal tubular epithelial cells (RPTCs). RPTCs were transfected with siRNA targeting p53 or negative siRNA (siNeg) and treated with MM102 (50 μM) for 1 h and then exposed to CP (20 μM) for an additional 24 h. Cell lysates were prepared and subjected to immunoblot analysis with antibodies against phospho-p53 (p-p53), p53, p21, cleaved caspase3 (C-cas3), E-cad (**A**), and MLL1, WDR5, H3K4me3, and histone H3 (**H**). p-p53 (**B**) was normalized with p53, p53 (**C**), p21 (**D**), E-cad (**F**), and C-cas3 (**G**) were normalized with GAPDH. MLL1 (**I**) and WDR5 (**J**) were normalized with GAPDH, respectively; H3K4me3 (**K**) was normalized with histone H3. Signal obtained from the control (Ctrl) sample was set as 1. **E** RPTCs were untreated or treated for 24 h with CP (20 μM) in the presence or absence of PFA (10 μM) with or without MM102 (50 μM). Cell viability was shown. Values are mean ± SD. A representative result from at least three experiments was shown. *P < 0.05, **P < 0.01, ***P < 0.001. ns means not significant.

We proceeded to examine the role of p53 in cisplatin-induced AKI. p53 knockdown with p53 siRNA (siP53) significantly reduced p53 protein levels (Fig. 6A, B, C) and p21 (Fig. 6A, D); siP53 alone abolished cisplatin-induced C-cas3; combination of siP53 and MM102 had a mild cumulative effect of attenuating cisplatin-triggered apoptosis, suggesting that besides MLL1/WDR5, p53 may also trigger other signaling pathways to induce cell death. Interestingly, siP53 treatment also restored the expression level of E-cadherin (Fig. 6A, E), suggesting that the cisplatin-induced downregulation of E-cadherin is p53-dependent.

In addition, we explored the relation between p53 and MLL1/WDR5 and H3K4me3. Figure 6H–K demonstrated that siP53 had no effect on the expression of MLL1 and WDR5 and their methyltransferase activity (as indicated by expression of H3K4me3), suggesting that p53 acts downstream of MLL1/WDR5 to rescue cisplatin-treated RPTCs from apoptosis.

We further used p53 inhibitor PFA [23] to determine the effect of p53 in cisplatin-induced apoptosis. Supplemental Fig. 4 showed that PFA alone abolished cisplatin-induced C-cas3; combination of PFA and MM102 had a cumulative effect on attenuating cisplatin-triggered apoptosis. The protective effect of PFA in RPTCs was also confirmed by measuring cell viability (Fig. 6G). Moreover, PFA treatment restored the expression level of E-cadherin (Supplemental Fig. 4). PFA exhibited little effect on the expression of p53 and E-cadherin or cell death in RPTCs without cisplatin exposure. PFA had no effect on the expression of MLL1/WDR5 and H3K4me3, either, further suggesting that p53 may act downstream of MLL1/WDR5 to mediate apoptosis.

Collectively, these results suggest that cisplatin may mediate nephrotoxicity through a MLL1/WDR5-p53-E-cadherin pathway.

### Inhibition of MLL1 or WDR5 blocks cisplatin-induced phosphorylation of ATR and ATM, and formation of γ-H2AX in vitro and in vivo

**Fig. 7:**
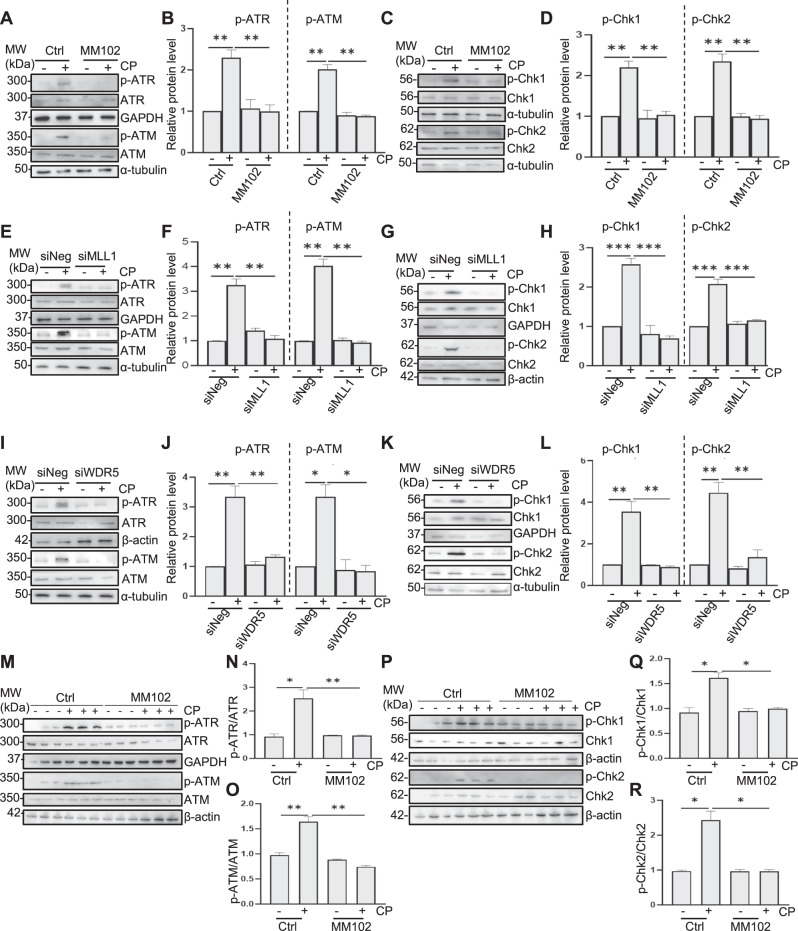
Inhibition of MLL1/WDR5 with MM102 or siRNA-mediated silencing of MLL1 or WDR5 blocks cisplatin (CP)-induced phosphorylation of ATM, ATR, Chk1, and Chk2 in cultured murine renal proximal tubular epithelial cells (RPTCs) and in kidneys. RPTCs were treated with MM102 (50 μM) or transfected with siRNA targeting MLL1 or WDR5 and then exposed to CP (20 μM) for an additional 24 h. Mice were intraperitoneally injected with MM102 (15 mg/kg) 2 h before the CP (20 mg/kg) injection, and then given the same dose daily for three consecutive days. All the mice were euthanized 72 h after CP injection. **A**, **E**, **I** Representative immunoblots for ATR, phosphorylated ATR (p-ATR), ATM, and phosphorylated ATM (p-ATM) in RPTCs. **B**, **F**, **J** Expression levels of p-ATR and p-ATM levels were quantified by densitometry and normalized with total ATR and ATM, respectively. **C**, **G**, **K** Representative immunoblots for phosphorylated Chk1 (p-Chk1), Chk1, phosphorylated Chk2 (p-Chk2), and Chk2 in RPTCs. Expression levels of those proteins were quantified by densitometry; p-Chk1 and p-Chk2 were normalized with total Chk1 and Chk2, respectively (**D**, **H**, **L**). Expression of p-ATR, ATR, p-ATM, ATM (**M**–**O**) and Chk1, Chk2, p-Chk1, and p-Chk2 (**P**–**R**) in mice were quantified by densitometry and normalized as indicated. Values are mean ± SD. A representative result from at least three experiments was shown. *P < 0.05, **P < 0.01, ***P < 0.001.

To explore whether DNA damage and DDR are involved in MLL1/WDR5-p53-mediated apoptosis, we examined the effect of MLL1/WDR5 inhibition on the phosphorylation and expression of ATM, ATR (recruited and activated by DNA double-strand breaks), and ‘transducer’ protein Chk1 and Chk2 [1]. As shown in Fig. 7, cisplatin treatment induced phosphorylation of ATR, ATM, Chk1, and Chk2, while MM102 treatment (Fig. 7A–D) and MLL1 (Fig. 7E–H) or WDR5 (Fig. 7I–L) silencing reduced phosphorylation to base levels. Similarly, MM102 treatment inhibited cisplatin-induced phosphorylation of ATR (Fig. 7M, N), ATM (Fig. 7M, O), Chk1 (Fig. 7P, Q), and Chk2 (Fig. 7P, R) in murine kidneys.

We continued to explore whether MLL1/WDR5 would regulate γ-H2AX formation, a critical event for DDR-associated phosphorylation of ATR/ATM [2]. We demonstrated that γ-H2AX was upregulated in cultured RPTCs (Supplemental Fig. 5A, C, E)) and in kidneys (Supplemental Fig. 5G) after cisplatin treatment; MM102 attenuated the induction of γ-H2AX in vitro (Supplemental Fig. 5A, B) and in vivo (Supplemental Fig. 5G, H). Silencing MLL1 (Supplemental Fig. 5C, D) or WDR5 (Supplemental Fig. 5E, F) also inhibited cisplatin-induced expression of γ-H2AX.

Collectively, these data suggest that MLL1/WDR5 is crucial for triggering DDR, thereby initiating ATR/ATM-Chk-dependent p53 activation.

### Inhibition of MLL1 or WDR5 relieves cisplatin-induced cell cycle arrest in vitro and in vivo

**Fig. 8:**
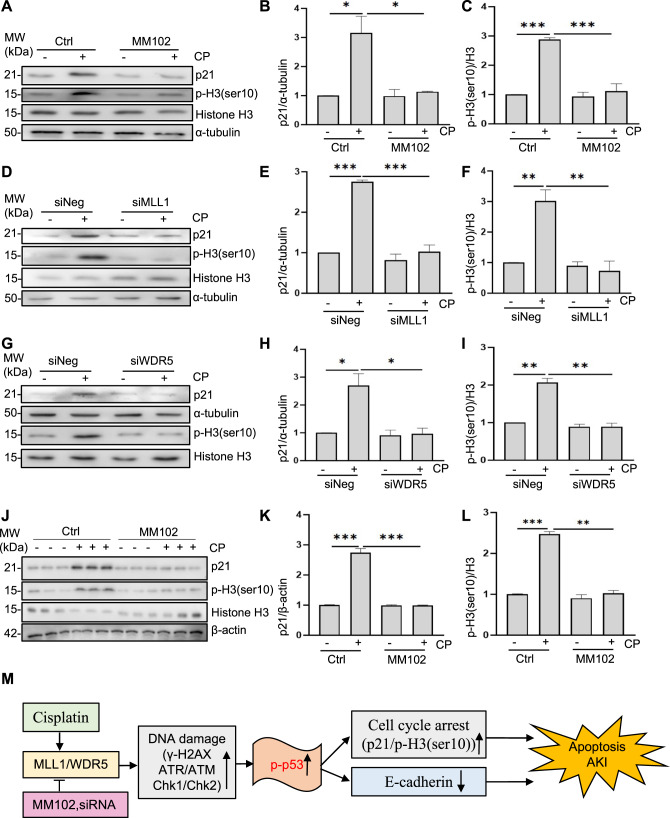
Blocking MLL1/ WDR5 inhibits cisplatin (CP)-induced cell cycle arrest in kidney of mice and in cultured murine renal proximal tubular epithelial cells (RPTCs). RPTCs were treated with MM102 (50 μM) or transfected with siRNA targeting MLL1 or WDR5 and then exposed to CP (20 μM) for an additional 24 h. Mice were intraperitoneally injected with MM102 (15 mg/kg) 2 h before CP (20 mg/kg) injection, and then given daily for three consecutive days. All the mice were euthanized 72 h after CP injection. The bands presented show Western blotting results for p21, phosphor-histone H3 on serine 10 (p-H3 (ser10)), histone H3 and references in vitro (**A**, **D**, **G**) and in vivo (**J**). **B**, **C**, **E**, **F**, **H**, **I**, **K**, **L** The graphs show p21 levels normalized to α-tubulin or β-actin, and p-H3 (ser10) normalized to H3. Values are mean ± SD. A representative result from at least three experiments was shown. *P < 0.05, **P < 0.01, ***P < 0.001. **M** Schematic diagram for the role of MLL1/WDR5 in CP-induced renal tubular cell apoptosis and acute kidney injury (AKI).

Cisplatin can induce chromatin condensation and cell cycle arrest during apoptosis [24]. To determine the role of MLL1/WDR5 in the cell cycle, we examined the expression of p21 and p-H3 (ser10) in kidneys and in cultured RPTCs. Expression of p21 (a downstream target of p53 involved in regulating cell cycle) and p-H3 (ser10) (phosphorylation of histone H3 on serine 10, a hallmark for cell cycle arrested at G2/M phase) was enhanced in cisplatin-treated RPTCs (Fig. 8A–I) and in mice (Fig. 8J–L), suggesting that cell cycle was arrested; MM102 treatment (Fig. 8A–C, J–L) and knockdown of MLL1 or WDR5 (Fig. 8D–I) decreased the cisplatin-induced upregulation of p21 and p-H3 (ser10). Taken together, these data suggest that MLL1/WDR5 is essential in driving cell cycle arrest during cisplatin-induced AKI.

## Discussion

In this study, we demonstrate that administration of MM102 improved renal function and attenuated pathological changes in cisplatin-treated mice as well as protected against cisplatin-induced RPTCs apoptosis in vitro. Meanwhile, MM102 inhibited p53 phosphorylation and restored E-cadherin expression. Our in vitro data also reveal that MLL1 activation contributes to cisplatin-induced RPTCs death via p53-dependent repression of E-cadherin. Furthermore, MLL1 is required for activation of key upstream regulators (ATR/ATM, Chk1, Chk2, and H2AX) of the cellular response to DNA damage necessary for activating p53 and expressing its target genes to mediate apoptosis and cell-cycle arrest. As such, these findings suggest that MLL1/WDR5 is actively involved in the early response to DNA damage following cisplatin exposure; it transduces the DDR signal to the associated kinases and then regulates p53 activation and subsequent E-cadherin depression and cell cycle arrest (Fig. 8M).

The present study demonstrates that MLL1/WDR5 is critically involved in the activation of ATR and ATM kinases by cisplatin in RPTCs and the kidney. This is evidenced by our findings that disruption of MLL1/WDR5 by MM102 or siRNA-mediated silencing of MLL1 or WDR5 inhibited ATR and ATM phosphorylation, blocked the formation of H2AX, a marker of DNA damage, and suppressed phosphorylation of Chk1 and Chk2, two downstream kinases of ATR and ATM. It is still poorly understood how MLL1/WDR5 regulates the DDR signaling network and regulates ATR/ATM activation. As ATR and ATM are activated via diverse protein complexes such as MRE11-RAD50-NBS1 [25] and are subjected to epigenetic regulation [26–28], it is possible that MLL1/WDR5 plays a role in the regulation of MRE11-RAD50-NBS1 or other activating complexes via methylation of H3K4 in response to DNA damage. It has been reported that the MLL family can be recruited to activate transcriptional factors to initiate oncogenic DNA damage and apoptosis during cell division [29, 30]. But MLL1 may directly regulate ATR or ATM through methylation of non-histone proteins. In support of this hypothesis, the MLL1/WDR5 complex has been reported to directly methylate the transcription factor SOX2 at lysine 42, triggering degradation of SOX2 [31]. SETD1A, another histone methyltransferases of SET1/MLL family, was found to mono-methylate Yes-associated protein at K342 to promote its activation [32]. Thus, a direct role of MLL1 in regulating ATR and/or ATM as well as their associated regulators is worthy of future exploration.

p53 is known as a major downstream effector of DDR during cisplatin-induced AKI [33, 34]. We found that inhibition of MLL1/WDR5 inhibited cisplatin-induced apoptosis in vivo and in vitro; blocking p53 also resulted in the similar inhibitory effect on apoptosis. These data, together with the effect of MLL1/WDR5 inhibition on the phosphorylation of p53 and its upstream activators (ATR/ATM, Chk1/Chk2), suggest that MLL1/WDR5 contributes to renal tubular apoptosis primarily via a p53-dependent activation of caspase-3. Cisplatin was also reported to induce tubular cell apoptosis through a p53-independent pathway involved in the activation of c-Abl [35, 36]. cAbl can be activated by ATM-mediated phosphorylation during cisplatin treatment [36]. We observed that pharmacological inhibition of MLL1/WDR5 or siRNA-mediated silencing of MLL1 or WDR5 abrogated ATM phosphorylation, implying that MLL1/WDR5 may also signal to ATM to trigger apoptosis via a p53-independent mechanism. This hypothesis needs to be determined by using p53 knockout mice in the future.

Our data suggest that E-cadherin downregulation is secondary to MLL1/WDR5-p53 activation during cisplatin-induced AKI and apoptosis. This is supported by our observations that: (1) Cisplatin-associated AKI was accompanied by decreased E-cadherin expression while MLL1/WDR5 inhibition resumed E-cadherin expression and renoprotection; (2) Inhibition of MLL1/WDR5-mediated p53 phosphorylation promoted cell survival and retained E-cadherin expression in cultured RPTCs exposed to cisplatin; (3) Silencing E-cadherin counteracted the protective effect of MLL1/WDR5 inhibition. Since application of MM102 and p53 siRNA individually or in combination restored E-cadherin expression to a similar degree, we suggest that MLL1/WDR5 and p53 relay in the same pathway to regulate E-cadherin expression. As E-cadherin expression is critical for maintaining tubule integrity and protecting against cell death [13, 24, 37], loss of E-cadherin would be an important mechanism by which MLL1/WDR5-p53 contributes to renal cell death following cisplatin treatment [14].

It remains unclear how MLL1/WDR5-p53 triggers E-cadherin downregulation in RPECs. E-cadherin expression is known to be regulated by both transcriptional and post-translational mechanisms [12, 15]. In tumor cells, p53 can transcriptionally promote E-cadherin expression by directly targeting the CDH1 (coding E-cadherin) promoter [38] or indirectly reducing DNMT1-mediated CDH1 promoter methylation [10, 11]. However, we found that cisplatin-induced p53 activation led to loss of E-cadherin in RPTCs. As such, it is less likely that p53 directly regulates E-cadherin expression. Nevertheless, E-cadherin transcription is also regulated by other transcription factors, like SNAIL, SLUG, ZEB1/2, and Twist1/2 [39], and activation of these transcriptional factors suppresses E-cadherin expression [39]. Thus, it will be interesting to explore whether p53 is able to coordinate with these transcriptional factors to reduce E-cadherin expression in RPECs following cisplatin treatment. On the other hand, there is a possibility that one or more post-translational mechanisms are coupled to E-cadherin loss in RPTC with cisplatin exposure. In this regard, E-cadherin can be degraded by several families of proteases, including caspases [40]. Our data show that cisplatin-induced activation of MLL1/WDR5-p53 pathway leads to caspase-3 cleavage, supporting this hypothesis.

DDR triggers not only cell death, but also DNA repair, cell cycle arrest, and senescence. Although DNA repair and cell cycle arrest may help renal cell survival and promote renal regeneration in the early stage when the kidney is subject to sublethal injury, incomplete or maladaptive repair of the kidney frequently occurs in severely injured kidneys, resulting in chronic pathologies characterized by G2/M phase cell cycle arrest, tubular atrophy and interstitial fibrosis [41, 42]. We observed increased expression of p21 and p-H3 (ser10) in the kidney and RPTCs following cisplatin exposure, while inhibition of MLL1/WDR5 reversed these responses. Thus, MLL1/WDR5 may also participate in the long-term sequelae of AKI. In agreement with our data, a recent study showed that MLL1/WDR5 inhibition ameliorates ischemia/reperfusion-induced renal senescence in mice [20].

In summary, this study demonstrates that MLL1/WDR5 promotes cisplatin-induced nephrotoxicity by regulating p53-mediated E-cadherin repression. The beneficial effect of MM102 is associated with inhibition of DNA damage-initiated apoptosis and cell cycle arrest of RPTCs. Targeting MLL1/WDR5 might have therapeutic potential to prevent and treat AKI induced by cisplatin.

## Materials and methods

### Reagents and antibodies

MM102 (HY-12220A) and PFA (HY-15484) were purchased from MedChemExpress (NJ, USA). Cisplatin was purchased from the Pharmacy of Rhode Island Hospital (NDC 0703-5747-11, RI, USA). Antibodies to C-cas3 (9664), MLL1 (14197), p53 (2524), p-p53 (ser15) (9284), ATM (2873), p-ATM (4526), ATR (2790), p-ATR (2853), Chk1 (2360), p-Chk1 (2348), Chk2 (2662), p-Chk2 (2661), and RIPA lysis buffer (9806) were purchased from Cell Signaling Technology (MA, USA). Antibodies to WDR5 (ab56919), histone H3 (ab1791), histone H2AX (ab124781), and γ-H2AX (ab81299) were from Abcam (MA, USA). E-cadherin was bought from Proteintech (20874-1-AP, IL, USA). Antibody to NGAL was purchased from R&D systems (AF1857, MN, USA). MLL1 siRNA (Assay ID 501621), Lipofectamine 2000 (11668019), and SuperSignal chemiluminescent substrate (34580) were from Thermo Fisher Scientific (MA, USA). H3K4me3 antibody (07-473), TUNEL Kit (11684795910), CCK8 (96992), DAPI (D9542), Creatinine Assay Kit (6M01K06250), and Urea Assay Kit (7C08K03750) were purchased from Sigma-Aldrich (MO, USA). Klotho antibody (sc-515942), WDR5 siRNA (sc-61799), E-cadherin siRNA (sc-35243), p53 siRNA (sc-29436), and negative siRNA (sc-37007) were purchased from Santa Cruz Biotechnology (CA, USA).

### Animals models of AKI and treatment

Male C57BL/6J mice aged 6–8 weeks and weighing 20–25 g were purchased from the Jackson Laboratory (ME, USA). The mice were randomly divided into four groups: (1) control, (2) MM102, (3) cisplatin, and (4) MM102 plus cisplatin. Cisplatin was intraperitoneally injected at the dose of 20 mg/kg. MM102 (15 mg/kg) dissolved in solvent containing 10% DMSO and 90% corn oil was administered intraperitoneally 2 h before the cisplatin injection and then given daily for three consecutive days. The dose of MM102 was selected according to a previous report [20]. For the control and cisplatin-alone groups, mice were injected with an equivalent amount of solvent. Mice in the control and MM102 groups were injected with an equal volume of a normal saline solution. All the mice were euthanized 72 h after cisplatin injection. Blood samples and kidney tissues were collected for further analysis. All experimental protocols were performed according to the National Institutes of Health Guidelines on the Care and Use of Laboratory Animals and approved by the Lifespan Animal Welfare Committee. The authorization number for the use of laboratory animals is 5074-19.

### Renal function and histology analysis

Murine blood was collected to detect the level of Scr and BUN using the Creatinine Assay Kit and Urea Assay Kit, separately, according to the manufacturer’s instructions. For histology analysis, paraffin-embedded kidney tissues were cut into sections of 4 μm. PAS staining was conducted to evaluate renal injury in mice, according to Paller’s method [43], under microscopy at ×200 or ×600 optical magnification. Five fields were randomly observed, and morphological damage (epithelial necrosis, luminal necrotic debris, and tubular dilation) was quantified using the following scale: none = 0; <10% = 1; 11–25% = 2; 26–75% = 3; and >75% = 4.

### Immunofluorescence staining

Immunofluorescence (IF) staining was carried out for histological examination, using 4 μm formalin-fixed paraffin-embedded sections. After deparaffinization, rehydration, antigen retrieval, and blocking, the sections were incubated with primary and secondary antibodies. Nuclei were stained with DAPI. Rabbit anti-MLL1 antibody, mouse anti klotho antibody, rabbit anti-p-p53, and goat anti-NGAL antibody were used. Fluorescence was visualized and photographed under fluorescence microscopy (Olympus, Tokyo, Japan) at magnifications ×400.

### Detection of apoptosis

Apoptosis was detected by TUNEL staining using the In Situ Cell Death Detection Kit, Fluorescein (Sigma-Aldrich, MO, USA) according to the manufacturer’s instructions. Positive cells were counted, and at least 10 fields per section for each sample were examined.

### Cell culture and treatment

Immortalized mouse renal proximal tubular cells (RPTCs) were kindly provided by Dr. Elsa Bella-Reuss (University of Texas Medical Branch, Galveston, TX). These cells were cultured in Dulbecco’s modified Eagle’s medium (DMEM) with F12 containing 5% fetal bovine serum (FBS) and 0.5% penicillin/streptomycin in an atmosphere of 5% CO_2_, and 95% air at 37 °C. When cells reached 60% confluence, they were exposed to cisplatin at 20 μM for various time periods in the presence or absence of MM102 as indicated in figures.

### Transfection of siRNA

Cells were seeded to 30–40% confluence in antibiotic-free medium and then were transfected with siRNA specific to MLL1, WDR5, E-cadherin, and p53 according to the manufacturer’s instructions. In parallel, negative siRNA (siNeg) was used as a control for off-target changes in RPTCs cells. Twenty-four hours after transfection, the medium was changed to normal culture medium, and cells were treated for subsequent experiments.

### Cell viability assay

CCK8 was used to measure cell viability, according to the manufacture’s instruction. Briefly, RPTCs were seeded into 96-well plates (2000 cells/well) and incubated at 37 °C. After treating with cisplatin in the presence or absence of inhibitors at various concentrations for different time periods, CCK8 reagent was added to each well and incubated at 37 °C for 1 h. Absorbance was measured at a wavelength of 450 nm using a microplate reader.

### Immunoblot analyses

Kidney tissue and cultured cells were extracted with RIPA lysis buffer containing a protease inhibitor cocktail (Sigma-Aldrich, MO, USA), and the protein concentration of cell lysates was measured by a BCA protein assay kit (Thermo Fisher Scientific, MA, USA). Equal amounts of protein (30 μg) were separated by SDS-PAGE gels and then transferred to PVDF membranes (Millipore Sigma, MA, USA). After blocking with 5% nonfat milk, membranes were incubated with a primary antibody and horseradish peroxidase-conjugated secondary antibodies (Millipore Sigma, MA, USA). Detected antibodies were visualized by Chemiluminescent Substrate (Thermo Fisher Scientific, MA, USA) and analyzed by ChemiDoc Imaging System (Bio-Rad, MA, USA).

### Immunoprecipitation

Immunoprecipitation was performed with rabbit monoclonal anti-MLL1 (14197; Cell Signaling Technology), mouse monoclonal anti-WDR5 (sc-393116; Santa Cruz Biotechnology) and normal rabbit IgG (2729; Cell Signaling Technology) After incubation overnight at 4 °C, the protein/antibody complex was added to protein A agarose beads slurry (Cell Signaling Technology). Following 4 h of incubation at 4 °C, the complex was washed with RIPA lysis buffer. Immuno-complexes were centrifuged and eluted by boiling in loading buffer. The eluted sample (15–30 µl) was loaded on SDS-PAGE gel, and the complex was incubated with a relative antibody via western blotting.

### Statistical analysis

Data are presented as mean ± SD of at least three independent experiments. One-way analysis of variance followed by Student’s *t*-test with Bonferroni correction was used for multiple comparisons. Differences between two groups were analyzed by Student’s *t*-test. *P* < 0.05 was considered statistically significant.

## Supplementary information

checklist

MLL1-cisplatin-Supplemental data-20220703

Original Data File

## Data Availability

The datasets used and/or analyzed during the current study are available from the corresponding author on reasonable request.
