# Supplementary material for: Histone methyltransferase MLL1 drives renal tubular cell apoptosis by p53-dependent repression of E-cadherin during cisplatin-induced acute kidney injury
Source: Cell Death Dis. 2022 Sep 6;13(9):770. doi: 10.1038/s41419-022-05104-0 (PMC9448773; doi:10.1038/s41419-022-05104-0)
Supplement: Supplementary file 2 — MLL1-cisplatin-Supplemental data-20220703 [file 41419_2022_5104_MOESM2_ESM.docx]

**
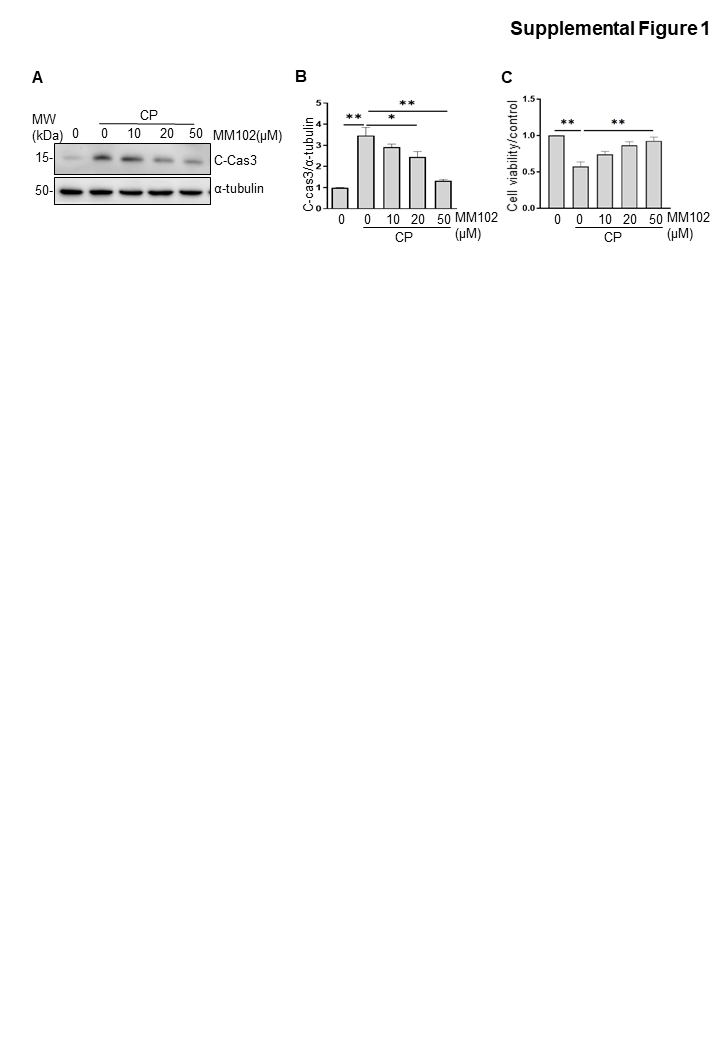
**

**Supplemental Figure 1. MM102 attenuates cisplatin (CP) associated apoptosis in a dose-dependent way in cultured murine proximal renal tubular epithelial cells (RPTCs).** RPTCs were treated with various concentrations of MM102 for 1 h and then exposed to CP (20 μM) for an additional 24 h. (A) Cell lysates were prepared and subjected to immunoblot analysis with antibodies against caspase3 cleavage (C-cas3) and α-tubulin. (B) The levels of C-cas3 were quantified by densitometry and normalized with α-tubulin. (C) Cell viability was detected 24 h after CP administered by cell counting kit 8 (CCK8) assay. Values are mean ± SD. A representative result from at least three experiments was shown. *P < 0.05, **P < 0.01.


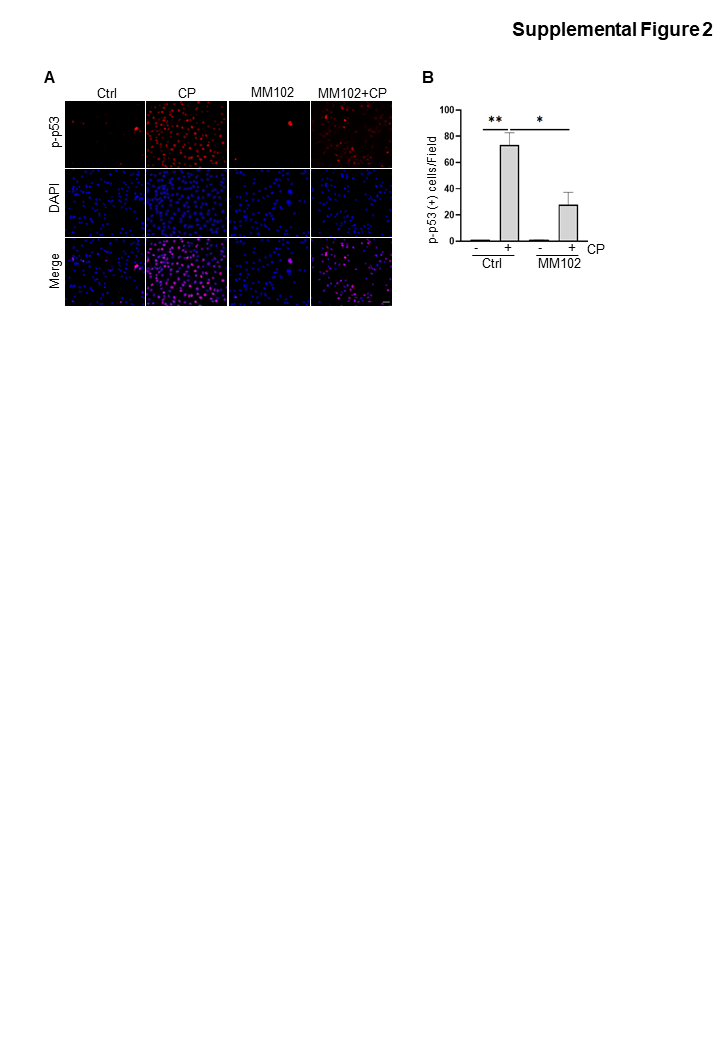
**Supplemental Figure 2. MM102 inhibits cisplatin (CP) induced p53 phosphorylation in cultured murine proximal renal tubular epithelial cells (RPTCs).** RPTCs were treated with MM102 (50 μM) for 1 hour and then exposed to CP (20 μM) for an additional 24 h. (A) Immunofluorescent staining for p-p53 is shown. Scale bars = 20 μm. (B) Quantification of the protein-positive area is shown in the right panel. Values are mean ± SD. A representative result from at least three experiments was shown. *P < 0.05, **P < 0.01


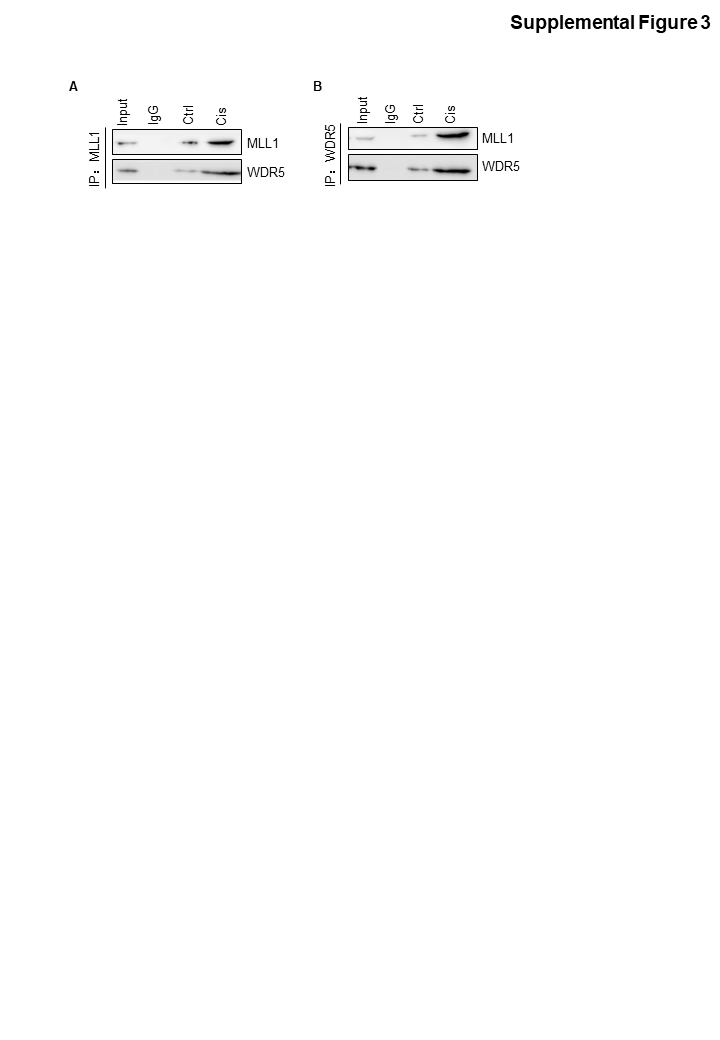
**Supplemental Figure 3. The interaction of MLL1 with WDR5 in renal proximal tubular cells (RPTC).** RPTCs were treated with MM102 (50 μM) for 1 hour and then exposed to CP (20 μM) for an additional 24 h. Cells were collected, and the cell lysates were then immunoprecipitated with an antibody against MLL1 (A) or WDR5 (B). The immunocomplexes were subjected to immunoblot analysis with antibodies to MLL1 and WDR5.

**
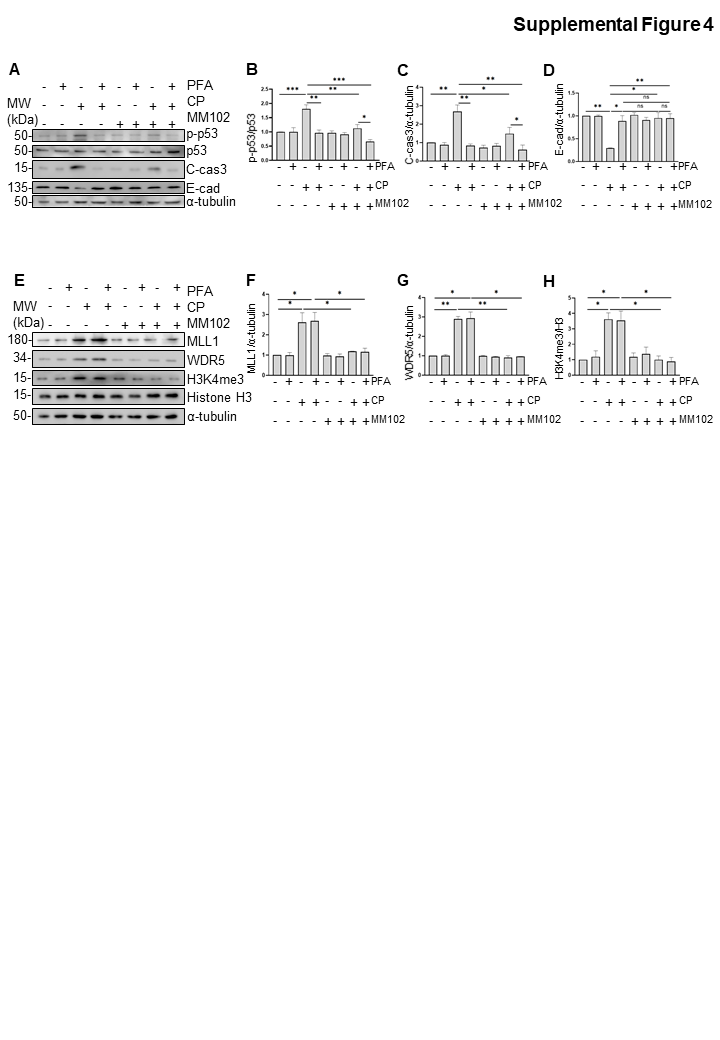
**

**Supplemental Figure 4. Inhibition of p53 with Pifithrin-α** (**PFA)** **diminishes cisplatin (CP)-induced apoptosis along with preservation of E-cadherin (E-cad) levels in murine renal proximal tubular epithelial cells (RPTCs).** RPTCs were untreated or treated for 24 h with CP (20 μM) in the presence or absence of PFA (10 μM) with or without MM102 (50 μM). Cell lysates were prepared and subjected to immunoblot analysis with antibodies against phospho-p53 (p-p53), p53, cleaved caspase3 (C-cas3), E-cad (A), and MLL1, WDR5, H3K4me3 and histone H3 (E). p-p53 (B) was normalized with p53, C-cas3 (C) and E-cad (D) were normalized with α-tubulin. MLL1 (F) and WDR5 (G) were normalized with α-tubulin; H3K4me3 (H) was normalized with histone H3. Signal obtained from the control (Ctrl) sample was set as 1. Values are mean ± SD. A representative result from at least three experiments was shown. *P < 0.05, **P < 0.01, ***P < 0.001. ns means not significant.


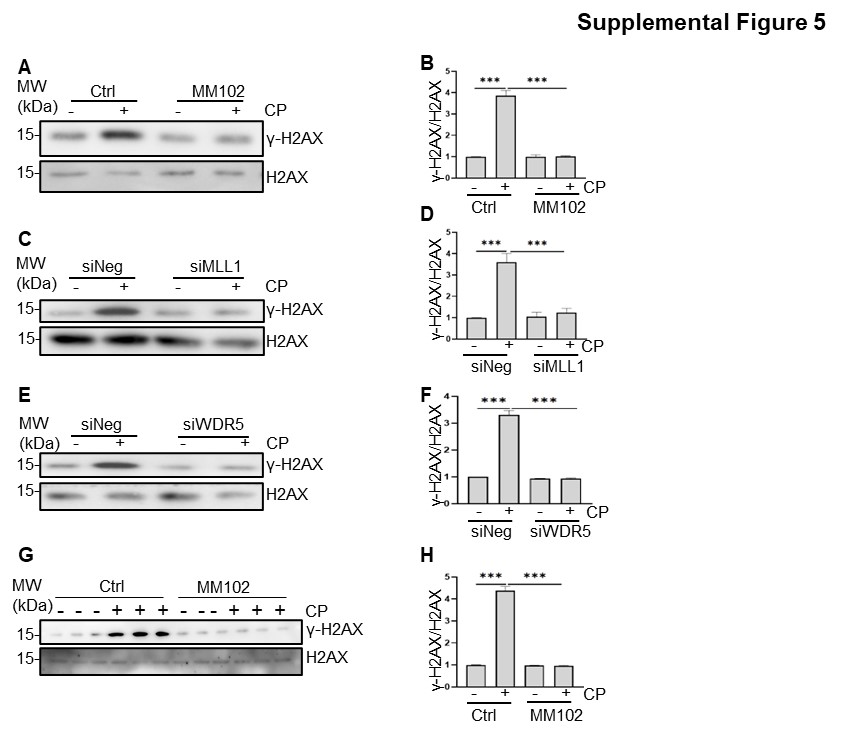
**Supplemental Figure 5. MLL1/ WDR5 is required for cisplatin (CP)-induced formation of γ-H2AX in kidney and in cultured murine proximal renal tubular epithelial cells (RPTCs).** RPTCs were treated with MM102 (50 μM) or transfected with siRNA targeting MLL1 or WDR5 and then exposed to CP (20 μM) for an additional 24 h. Mice were intraperitoneally injected with MM102 (15 mg/kg) 2 h before the CP (20 mg/kg) injection, and then given daily for three consecutive days. All the mice were euthanized 72 h after CP injection (n=5 for each group). The bands and graphs presented show Western blotting results for phospho-histone H2AX (γ-H2AX), H2AX in vitro (A-F) and in vivo (G, H). Values are mean ± SD. A representative result from at least three experiments was shown. ***P < 0.001.
