# Supplementary material for: Histone methyltransferase MLL1 drives renal tubular cell apoptosis by p53-dependent repression of E-cadherin during cisplatin-induced acute kidney injury
Source: Cell Death Dis. 2022 Sep 6;13(9):770. doi: 10.1038/s41419-022-05104-0 (PMC9448773; doi:10.1038/s41419-022-05104-0)
Supplement: Supplementary file 3 — Original Data File [file 41419_2022_5104_MOESM3_ESM.pptx]

## Slide 1
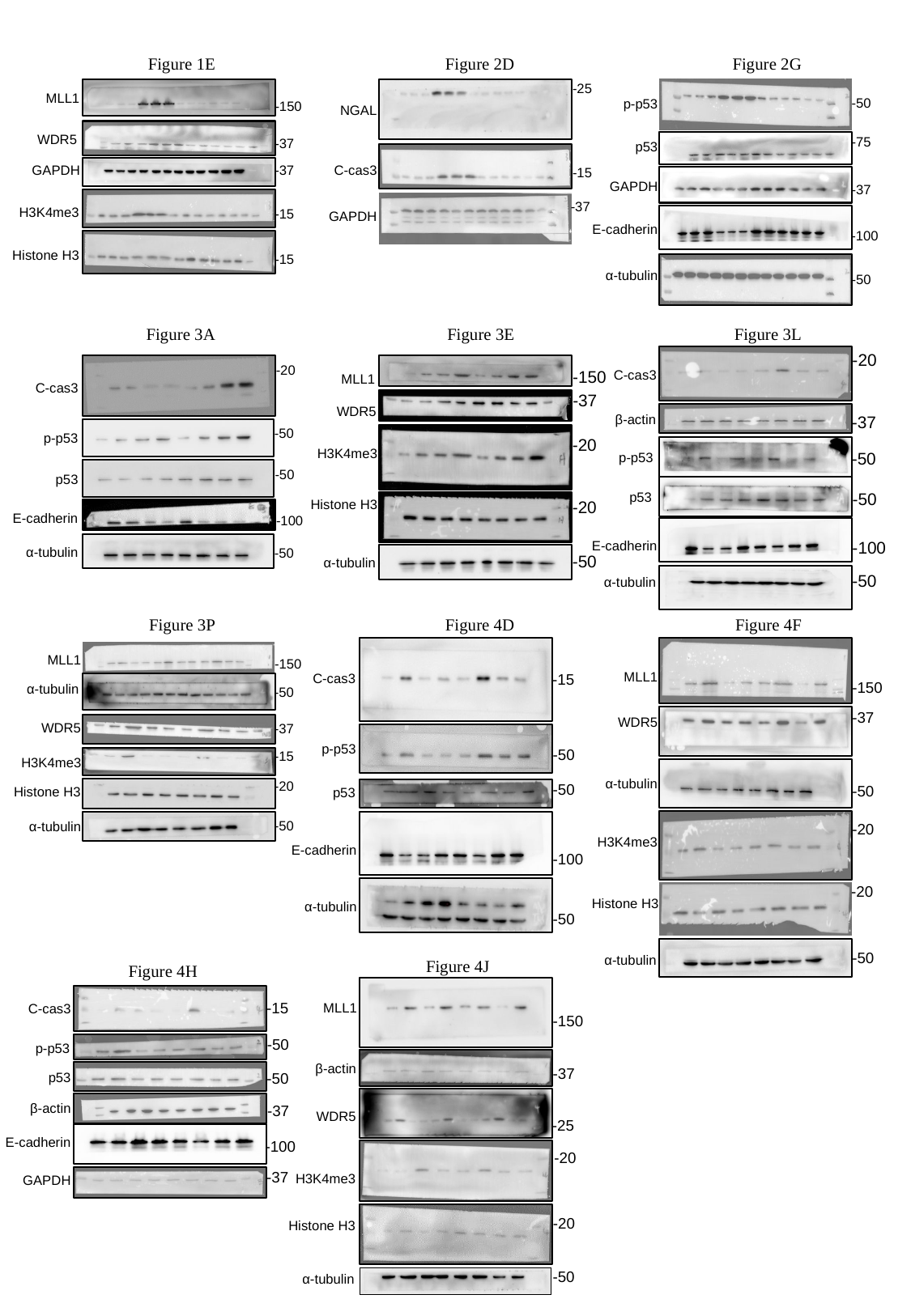

Figure 1E
Figure 2D
Figure 2G
-25
-15
-37
MLL1
WDR5
GAPDH
H3K4me3
Histone H3
-50
-75
-37
-100
-50
p-p53
p53
GAPDH
E-cadherin
α-tubulin
-150
-37
-37
-15
-15
 NGAL
 C-cas3
 GAPDH
Figure 3A
Figure 3E
Figure 3L
-20
-37
-50
-50
-100
-50
-20
-50
-50
-100
-50
-150
-37
-20
-20
-50
 C-cas3
β-actin
p-p53
p53
E-cadherin
α-tubulin
MLL1
WDR5
H3K4me3
 Histone H3
α-tubulin
C-cas3
p-p53
p53
E-cadherin
α-tubulin
Figure 3P
Figure 4D
Figure 4F
MLL1
α-tubulin
WDR5
H3K4me3
Histone H3
α-tubulin
-150
-50
-37
-15
-20
-50
MLL1
WDR5
α-tubulin
H3K4me3
Histone H3
α-tubulin
C-cas3
p-p53
p53
E-cadherin
α-tubulin
-15
-50
-50
-100
-50
-150
-37
-50
-20
-20
-50
Figure 4J
Figure 4H
-15
-50
-50
-37
-100
-37
MLL1
β-actin
WDR5
H3K4me3
Histone H3
α-tubulin
C-cas3
p-p53
p53
β-actin
E-cadherin
GAPDH
-150
-37
-25
-20
-20
-50

## Slide 2
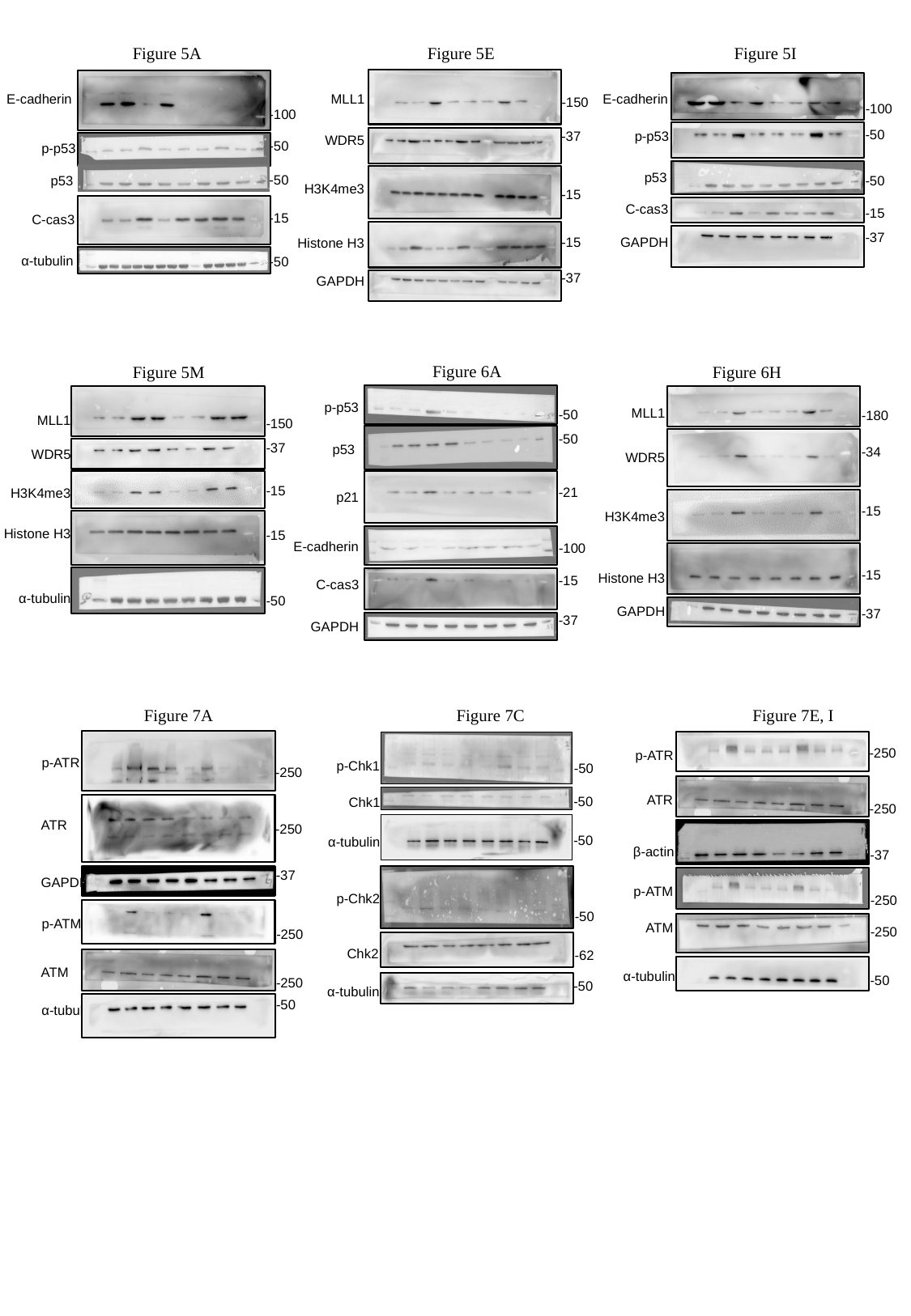

Figure 5A
Figure 5E
Figure 5I
E-cadherin
p-p53
p53
C-cas3
α-tubulin
E-cadherin
p-p53
p53
C-cas3
GAPDH
MLL1
WDR5
H3K4me3
Histone H3
GAPDH
-150
-37
-15
-15
-37
-100
-50
-50
-15
-37
-100
-50
-50
-15
-50
Figure 6A
Figure 5M
Figure 6H
p-p53
p53
p21
E-cadherin
C-cas3
GAPDH
MLL1
WDR5
H3K4me3
Histone H3
GAPDH
-50
-50
-21
-100
-15
-37
-180
-34
-15
-15
-37
MLL1
WDR5
H3K4me3
Histone H3
α-tubulin
-150
-37
-15
-15
-50
Figure 7A
Figure 7C
Figure 7E, I
-250
-250
-37
-250
-250
-50
p-ATR
ATR
β-actin
p-ATM
ATM
α-tubulin
p-ATR
ATR
GAPDH
p-ATM
ATM
α-tubulin
p-Chk1
Chk1
α-tubulin
p-Chk2
Chk2
α-tubulin
-50
-50
-50
-50
-62
-50
-250
-250
-37
-250
-250
-50

## Slide 3
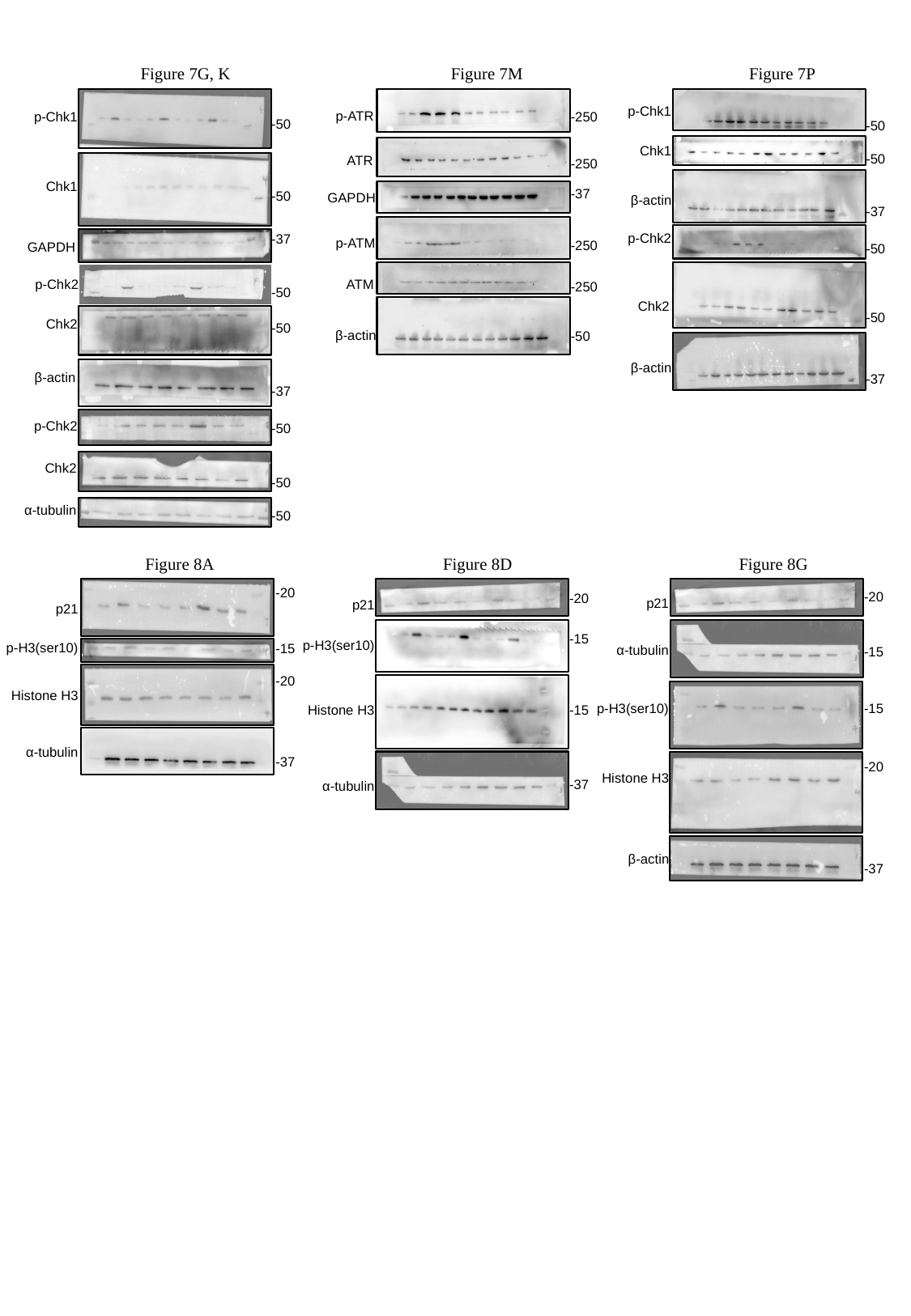

Figure 7G, K
Figure 7M
Figure 7P
p-Chk1
Chk1
β-actin
p-Chk2
Chk2
β-actin
p-ATR
ATR
GAPDH
p-ATM
ATM
β-actin
p-Chk1
Chk1
GAPDH
p-Chk2
Chk2
β-actin
p-Chk2
Chk2
α-tubulin
-250
-250
-37
-250
-250
-50
-50
-50
-37
-50
-50
-37
-50
-50
-50
-50
-50
-37
-50
-50
-37
Figure 8A
Figure 8D
Figure 8G
-20
-15
-20
-37
-20
-15
-15
-20
-37
-20
-15
-15
-37
p21
α-tubulin
p-H3(ser10)
Histone H3
β-actin
p21
p-H3(ser10)
Histone H3
α-tubulin
p21
p-H3(ser10)
Histone H3
α-tubulin

## Slide 4
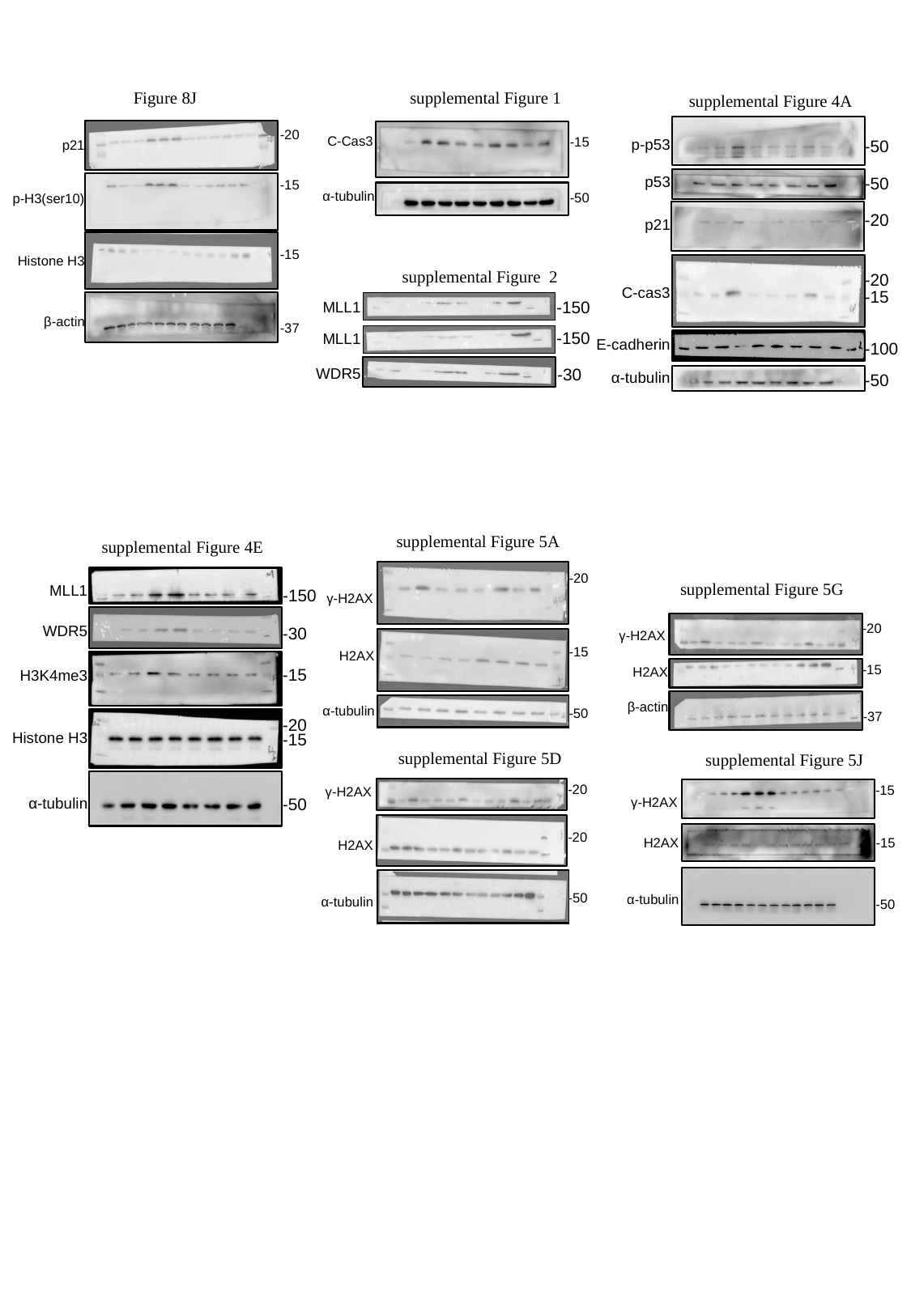

Figure 8J
supplemental Figure 1
supplemental Figure 4A
-20
-15
-15
-37
C-Cas3
α-tubulin
-15
-50
p-p53
p53
p21
C-cas3
E-cadherin
α-tubulin
-50
-50
-20
-20
-15
-100
-50
p21
p-H3(ser10)
Histone H3
β-actin
supplemental Figure 2
-150
MLL1
-150
MLL1
WDR5
-30
supplemental Figure 5A
supplemental Figure 4E
-20
-15
-50
supplemental Figure 5G
MLL1
WDR5
H3K4me3
Histone H3
α-tubulin
-150
-30
-15
-20
-15
-50
γ-H2AX
H2AX
α-tubulin
-20
-15
-37
γ-H2AX
H2AX
β-actin
supplemental Figure 5D
supplemental Figure 5J
-20
-20
-50
-15
-15
-50
γ-H2AX
H2AX
α-tubulin
γ-H2AX
H2AX
α-tubulin
